# Association Between Undernutrition and the Number of Molar Occlusions in Older Persons Requiring Care in Long-Term Care Insurance Facilities

**DOI:** 10.3390/nu17040630

**Published:** 2025-02-10

**Authors:** Koji Takahashi, Yutaka Watanabe, Takuma Okumura, Yasushi Tamada, Misuzu Sato, Masanori Iwasaki, Maki Shirobe, Hirohiko Hirano, Yoshihiro Kugimiya, Masako Kishima, Kayoko Ito, Yasuyuki Iwasa, Yoshihiko Watanabe, Shinsuke Mizutani, Kazuharu Nakagawa, Shigekazu Komoto, Yutaka Yamazaki

**Affiliations:** 1 Gerodontology, Department of Oral Health Science, Faculty of Dental Medicine, Hokkaido University, Sapporo 060-8586, Japan; osdltd@icloud.com (K.T.); t.okumura@den.hokudai.ac.jp (T.O.); tamada@den.hokudai.ac.jp (Y.T.); yutaka8@den.hokudai.ac.jp (Y.Y.); 2 Department of Preventive Dentistry, Faculty of Dental Medicine and Graduate School of Dental Medicine, Hokkaido University, Sapporo 060-8586, Japan; misuzu@den.hokudai.ac.jp (M.S.); iwasaki@den.hokudai.ac.jp (M.I.); 3 Tokyo Metropolitan Institute for Geriatrics and Gerontology, Tokyo 173-0015, Japan; mshirobe@tmig.or.jp (M.S.); h-hiro@gd5.so-net.ne.jp (H.H.); 4 Department of Dentistry and Oral Surgery, National Center for Geriatrics and Gerontology, Obu 474-8511, Japan; kugimiyay@ncgg.go.jp; 5 Department of Dentistry, Wakakusa-Tatsuma Rehabilitation Hospital, Daito 574-0012, Japan; tdbqd500@yahoo.co.jp; 6 Oral Rehabilitation, Niigata University Medical and Dental Hospital, Niigata 951-8520, Japan; k-ito@dent.niigata-u.ac.jp; 7 Department of Dentistry, Haradoi Hospital, Fukuoka 813-8588, Japan; y_iwasa@haradoi-hospital.com; 8 Department of Healthcare Management, Tohoku Fukushi University, Sendai 981-8522, Japan; yoshiw@tfu.ac.jp; 9 Section of Geriatric Dentistry and Perioperative Medicine in Dentistry, Division of Maxillofacial Diagnostic and Surgical Sciences, Faculty of Dental Science, Kyushu University, Fukuoka 812-8582, Japan; mizutani@dent.kyushu-u.ac.jp; 10 OBT Research Center, Faculty of Dental Science, Kyushu University, Fukuoka 812-8582, Japan; 11 Department of Dysphagia Rehabilitation, Division of Gerontology and Gerodontology, Graduate School of Medical and Dental Sciences, Institute of Science Tokyo, Tokyo 113-8510, Japan; k.nakagawa.swal@tmd.ac.jp; 12 Department of Health Care Policy, Faculty of Medicine and Graduate School of Medicine, Hokkaido University, Hokkaido 060-8638, Japan; komoto@med.hokudai.ac.jp

**Keywords:** long-term care insurance facilities, malnutrition, occlusal support, older adults

## Abstract

**Background/Objectives**: Undernutrition increases the mortality risk in older persons requiring long-term care; further, it is associated with oral functions such as swallowing and chewing. Moreover, occlusion affects oral function and is crucially involved in nutritional intake. The present study aimed to examine the association between the number of molar occlusions and undernutrition according to body mass index (BMI) in older persons requiring long-term care. **Methods**: Japanese older persons requiring long-term care were categorized based on BMI (<20 kg/m^2^ vs. 20 kg/m^2^). We examined the association between undernutrition and the number of molar occlusions (one in each of the left and right premolars and molars, for a total of four). **Results**: Among 893 included participants, 440 (49.3%) had BMI < 20 kg/m^2^ and 453 (50.7%) had BMI > 20 kg/m^2^. Binomial logistic regression analysis revealed that BMI < 20 kg/m^2^ was significantly associated with increased number of molar occlusions (odds ratio: 0.52–0.70, 95% CI: 0.28–1.00). This indicated that a decrease in the number of molar occlusions was associated with malnutrition as determined through BMI in older persons requiring long-term care and residing in long-term care insurance facilities in Japan. **Conclusions**: Our findings suggest that maintaining occlusal support may help maintain nutritional status in older persons requiring long-term care.

## 1. Introduction

According to the “Population Estimates 2024 August Report” published by the Statistics Bureau of Japan, the proportion of people aged ≥ 65 years among the overall population is 29.3% in Japan and the highest in the world [1]. Accordingly, there has been a rapid increase in the number of older persons requiring long-term care and residents of long-term care insurance facilities who require a continuously high level of care [2]. Provision of professional oral hygiene management (OHM) in residents of long-term care facilities is associated with a decreased incidence of pneumonia [3], which suggests that maintenance and improvement of both oral hygiene and oral function can affect overall health. Furthermore, poor oral function has been associated with malnutrition [4]. Since malnutrition is associated with decreased quality of life (QoL) in older persons requiring long-term care [5], nutritional management in these individuals is becoming increasingly important [6,7,8]. However, nutritional disorders in older persons receiving long-term care are often underdiagnosed and undertreated, which highlights the need for routine nutritional screening as part of daily care [9].

Since malnutrition has been associated with oral functions such as swallowing [10], there is a presumed relationship between oral health and nutritional status in older persons requiring long-term care. However, a survey of older persons requiring long-term care in Japan found that despite 64.3% of them requiring dental care or oral health care, only 2.4% had actually received dental care within the past year [11]. Furthermore, a survey on services provided in geriatric health services facilities and long-term care medical clinics found that fewer than 60% of all residents received oral screening [12]. The lack of an established direct link between dental care and oral problems in older persons requiring long-term care could be attributed to the lack of screening indicators for oral problems [12].

Accordingly, the 2024 revision of medical care and long-term care fees in Japan recommended eight evaluation criteria for examinations performed by professionals other than dental professionals in the medical care field [13]. One of these evaluation items was the evaluation of occlusal support of the molars [13,14]. Among older persons requiring long-term care, loss of occlusal support in the molars has been associated with various systemic conditions, including suffocation accidents [15], dementia [16], Alzheimer’s disease [17], and fever [18]. Contrastingly, a decrease in occlusal support can result in occlusal problems such as tooth movement and occlusal interference [19,20]. Accordingly, occlusal support in the molars is considered a crucial factor in oral health. Furthermore, decreased chewing ability due to loss of molar occlusions has been shown to influence the choice of food [21]. Therefore, the loss of molars may lead to undernutrition due to a decrease in nutrient intake from the diet. Since the loss of molar occlusions can be addressed through prosthetic procedures such as dentures, appropriate prosthetic treatment may contribute towards the prevention of undernutrition in older persons requiring long-term care.

However, the relationship between occlusal support and nutritional status in older persons requiring long-term care remains unclear. We hypothesized that undernutrition is associated with a decreased number of molar occlusions in older persons residing in long-term care insurance facilities. The aim of this cross-sectional study was to investigate the association between molar malocclusion and undernutrition in terms of the body mass index (BMI) stratified (BMI < 20 kg/m^2^ and BMI ≥ 20 kg/m^2^) according to the Global Leadership Initiative on Malnutrition [22] in older adults residing in long-term care insurance facilities in Japan.

## 2. Materials and Methods

### 2.1. Study Design and Participants

This was a cross-sectional study of older persons who were residents of long-term care insurance facilities in Japan. In total, 986 older people aged ≥ 65 years were recruited from 29 long-term care insurance facilities located in 15 regions of Japan.

The members of the Special Committee of the Japanese Society of Geriatrics and Dentistry explained the study contents, verbally and in writing, to the directors and staff of long-term care insurance facilities with which they were working. Then, the study contents were explained, verbally and in writing, to the residents of nursing care facilities that cooperated with the study, following which written informed consent was obtained. When it was difficult to obtain consent from an individual because of a decline in cognitive function, written study details were sent to the family members, who then provided written consent. Questionnaire surveys and face-to-face surveys were conducted from November 2019 to March 2020 for individuals who agreed to participate. The questionnaires were mailed to long-term care insurance facilities staff in charge of the residents. For the face-to-face survey, trained investigators who used standardized survey criteria visited each facility, conducted the survey, and evaluated the results. This study was conducted in accordance with the Declaration of Helsinki and approved by the Ethics Committee of the Japan Geriatrics Society (protocol code 2018-1 and 17 October 2018) and the Hokkaido University Faculty of Dental Medicine (protocol code 2020-4 and 18 August 2020).

### 2.2. Survey Items

To conduct the survey, each member provided training about the survey to all nurses, dietitians, dentists, and dental hygienists at the facilities in order to standardize the evaluation criteria. The survey was distributed to each facility, and the nurses and dietitians collected data regarding the following items.

#### 2.2.1. Survey

##### Basic Information

Nurses and/or dietitians in charge of the participants collected the following variables from the residents’ records: age, sex, BMI, provision of OHM care services, methods of nutritional intake (regular diet, dysphagia diet, enteral nutrition), and medical history (aspiration pneumonia; cerebrovascular disease; diabetes; respiratory disease; cardiovascular disease; neoplastic disease, including cancer; Parkinson’s disease; neurological disease; depression; and dementia).

##### Performance in Activities of Daily Living (ADL) and Cognitive Functions

Nurses in charge of the participants assessed their performance in ADL using the Barthel Index (BI). The total score for each BI item ranged from 0 to 100, with higher scores indicating better ADL [23]. Cognitive function was assessed by nurses who received prior training regarding standardized assessment criteria from members using the Clinical Dementia Rating (CDR) as described by Morris et al. [24,25]. The final evaluation of CDR was performed using the CDR^®^ Dementia Staging Instrument calculator (Washington University, St. Louis, MO, USA) [26]. Higher grades indicated greater cognitive impairment.

##### Diet Styles

The diet styles were defined based on the Japanese Society of Dysphagia Rehabilitation’s code of Japanese dysphagia diet 2013, with those meeting the definition criteria being categorized as dysphagia diet [27]. The rest were classified as either regular diet or enteral nutrition based on the intake method.

##### Number of Molar Occlusions

The participants were examined by dentists and dental hygienists who received prior training regarding the standardized evaluation criteria. Bilateral premolars and molars (four pairs in total) were considered to have occlusal support if the same teeth were present in the upper and lower jaws or dentures (if the participant was wearing dentures). If at least one of the two premolars or two molars was in occlusion with its antagonist tooth, then occlusal support was considered present. For example, if there were two upper and two lower right premolars in occlusion, there were two pairs of occluding teeth, but the score was 1. If there were two upper right premolars and molars and two lower right premolars and molars in occlusion, there were four pairs of occluding teeth, but the score was 2.

### 2.3. Statistical Analyses

For comparative analysis, participants were divided into two groups: BMI < 20 kg/m^2^ and BMI ≥ 20 kg/m^2^, considering that BMI < 20 kg/m^2^ is considered to indicate malnutrition according to the Global Leadership Initiative on Malnutrition [22]. The Shapiro-Wilk test was performed on continuous variables to assess the normality of data distribution. Normally and non-normally distributed continuous variables were analyzed using a *t*-test and Mann-Whitney U test, respectively. Categorical variables were analyzed using a chi-square test.

The dependent variables were BMI < 20 kg/m^2^ and BMI ≥ 20 kg/m^2^, while the independent variables were the number of molar occlusions, age, sex, medical history (aspiration pneumonia, cerebrovascular disease, diabetes, respiratory disease, cardiovascular disease, neoplastic disease, Parkinson’s disease, neurological disease, depression), BI [10] (associated with BMI), CDR [10], OHM status [28], nutritional intake methods [10,29], and dysphagia [10,30]. All statistical analyses were performed using SPSS Statistics Ver.29 [30] (IBM, Armonk, NY, USA), with significance being set at <5% (*p* < 0.05).

## 3. Results

**Figure 1:**
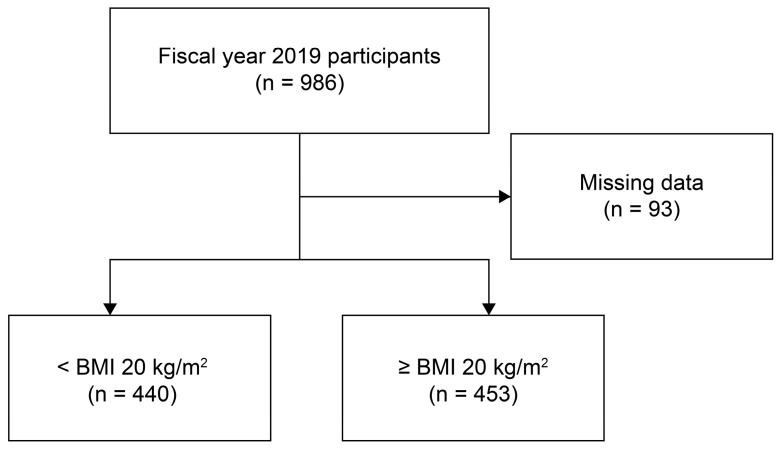
Study flowchart. A survey was conducted on 986 older persons requiring long-term care who resided in long-term care insurance facilities and agreed to participate in the study. After excluding 93 persons with missing necessary data, 893 were analyzed after stratification into BMI < 20 kg/m^2^ (440 persons) and BMI ≥ 20 kg/m^2^ (453 persons) groups.

Among 986 older persons requiring long-term care who resided in long-term care insurance facilities, we included 893 participants (mean age 86.8 ± 7.93 years, 174 men [19.5%], and 719 women [80.5%]), after excluding 93 participants with missing data (Figure 1). None of the participants had any implant prostheses.

Among the 893 included participants, 440 (49.3%) participants had BMI < 20 kg/m^2^ and 453 (50.7%) participants had BMI ≥ 20 kg/m^2^. BI scores were significantly higher in those with BMI ≥ 20 kg/m^2^ than in those with BMI < 20 kg/m^2^. Among categorical variables, there were significant between-group differences in CDR score, number of molar occlusions, methods of nutritional intake, dysphagia, and aspiration pneumonia (Table 1).

Binomial logistic regression analysis was performed to examine factors related to BMI, with BMI ≥ 20 kg/m^2^ and BMI < 20 kg/m^2^ as dependent variables. BMI < 20 kg/m^2^ was associated with a increase in the number of molar occlusions (odds ratio [OR]: 0.52–0.70, 95% confidence interval [CI]: 0.28–1.00), poor methods of nutritional intake (OR: 2.53–4.13, 95% CI: 1.78–9.61), absence of dysphagia (OR: 0.58, 95% CI: 0.38–0.89), and a history of Parkinson’s disease (OR: 2.12, 95% CI: 1.09–4.11) (Table 2).

## 4. Discussion

Our findings indicated that a decrease in the number of molar occlusions was associated with undernutrition, as determined by BMI, in older persons requiring long-term care. These findings suggest that restoration of occlusal support, for example through prosthetic denture treatment in cases of tooth loss, may help maintain nutritional status in older persons requiring long-term care. Our findings are consistent with previous reports suggesting that regular dental management for older persons requiring long-term care may reduce weight loss and prevent undernutrition [28]. Additionally, our findings indicate that the maintenance and restoration of molar occlusal support in regular dental management can help prevent undernutrition.

In addition to the number of molar occlusions, we observed a correlation of undernutrition in terms of BMI with dysphagia, Parkinson’s disease, and poor nutritional intake methods. Previous studies have demonstrated a relationship between dysphagia and weight loss among older persons requiring long-term care [31,32]. Similarly, another study demonstrated an association between low BMI and Parkinson’s disease [33]. A previous longitudinal study found that the maintenance of nutritional intake methods, specifically the feeding and swallowing function, was associated with maintenance of body weight [29]. Taken together, our findings are consistent with previous reports. Consistent with a previous report [10], we observed significant between-group differences in the BI and CDR. However, we observed no correlation of low BMI with age and OHM. Consistent with this finding, a previous study on routine dental management [28] demonstrated that despite there being a relationship between age and OHM in older persons requiring care who were on a regular diet, there was no significant relationship among individuals on a dysphagia diet. In addition to OHM aimed at improving oral hygiene, OHM also includes oral function management aimed at maintaining and improving functions such as chewing and swallowing. Accordingly, the primary objective of OHM for older persons requiring intensive long-term care who have difficulty maintaining and improving functions, such as swallowing and chewing, may shift more towards oral hygiene management.

A decrease in the number of occlusions between functional teeth, including present teeth, pontics, and dentures, is associated with poor nutritional intake methods in older persons who require long-term care [34]. Additionally, as previously noted, maintenance of nutritional intake methods is associated with maintenance of body weight [29]. These findings indicate that a decrease in the number of occlusal supports is associated with poor nutritional intake methods and dysphagia, resulting in reduced BMI. Given the relationship of occlusal support with dietary and nutrient intake [35,36], as well as that of BMI with dietary intake and nutrient quality [37,38], decreased occlusal support may have led to nutrient deficiencies, which in turn affected BMI.

Decreased occlusal support is associated with decreased fiber intake [36]. Dietary fiber and other nutrients act on the intestinal bacteria as prebiotics; moreover, imbalance of intestinal bacteria contributes to digestive problems [39]. Additionally, a low number of teeth results in poor chewing ability [40], which is associated with indigestion [41]. Therefore, intestinal bacteria and chewing ability may affect the BMI of older persons requiring long-term care due to poor digestion of food and nutrient absorption.

In the present study, BMI was used as an indicator of nutritional status since it can be routinely measured by caregivers. Weight loss is considered a very crucial indicator of undernutrition [42], and low BMI is associated with increased short-term mortality rates in the older population [43]. Therefore, maintaining weight among residents of long-term care facilities is important for maintaining overall health. Therefore, we used BMI as an indicator of nutritional status for the purpose of our study.

Furthermore, we used BMI < 20 kg/m^2^ as the threshold for undernutrition. This was based on the Global Leadership Initiative on Malnutrition [22] established in 2018 as a global definition of undernutrition, as well as the criteria for undernutrition for individuals aged ≥ 65 years as defined by the third term of the Health Japan 21 Basic Policy published by the Japanese Ministry of Health, Labour and Welfare in 2023. Similarly, an Italian longitudinal study of 3110 randomly selected adults aged 65–84 years demonstrated that BMI < 20 kg/m^2^ is a reliable threshold for defining underweight older adults at a high risk of short-term mortality [44]. In addition, Wirth et al. conducted a study of 10,298 residents in 191 nursing homes in 13 countries (“Nutrition Day in Nursing Homes Project” (2007–2012)) and found that a low BMI of < 20 kg/m^2^ and weight loss of ≥ 5 kg within a year were important factors associated with 6-month mortality [45].

Most studies on nutrition in older persons requiring long-term care have applied the Mini Nutritional Assessment^®^ (MNA^®^) to assess nutritional status [46,47]. However, non-trained professionals are required to examine physical conditions, which can be time-consuming given the large number of variables to assess. Therefore, this assessment may be unsuitable in the settings of long-term care insurance facilities hosting patients with poor cognitive functions. Furthermore, most older persons requiring long-term care can be categorized in the poor risk group based on the three-point MNA scale for nutritional assessment, which impedes accurate assessment of nutritional status [48]. On the other hand, since BMI is routinely measured in Japanese long-term care settings, it does not require specific skills to measure and is an objective index with little bias. Accordingly, we considered BMI to be a suitable index of nutritional status in older persons requiring intensive long-term care who reside in long-term care insurance facilities.

Regarding the sample size, previous studies on older persons requiring long-term care include a cross-sectional study on the nutritional status and sarcopenia of 386 older persons residing in special nursing homes in China [49], a cross-sectional study on 322 older persons requiring long-term care in Japan that examined the association between denture wear and nutritional status [50], and our previous cross-sectional study examining the association between occlusal support and diet styles in 888 Japanese older persons requiring long-term care [34]. Accordingly, the present study included a sufficient sample size greater than previous studies given that we included 986 older persons requiring long-term care in 29 long-term care insurance facilities within 15 Japanese regions.

The mean age and female proportion in our study were 86.8 years and 80.5%, respectively. Furthermore, the mean BI was 30, the proportion of patients with cognitive impairment (CDR ≥ 1) was 89.5%; 53.0% had suspected dysphagia or higher, and 32.5% had no occlusions. These characteristics are similar to that of a cohort of a previous study on occlusal support, dysphagia, malnutrition, and ADL in older persons requiring long-term care in Japan [31]. In this previous study, the mean age and female proportion was 83 years and 67%, respectively; 39% had no occlusal support, and the mean BI was 30. In a German study of nursing home residents [51], the mean age and female proportion were 84.1 years and 72%, respectively; further, 47% had dementia and 48% were edentulous. Generally, participants in Japanese studies tend to be older than those in studies conducted outside of Japan; moreover, a larger proportion of Japanese participants are cognitively impaired. According to the October 2024 Population Estimates released by the National Bureau of Statistics [52], Japan has the highest rate of aging population, with 29.3% of the population being aged ≥ 65 years. Furthermore, the prevalence of dementia is high in Japan [53]. Accordingly, our participants can be generally considered to be representative of long-term insurance facility residents in Japan and comparable to those in other countries whose population is expected to continue to age.

Related to this research, a study in Germany reported that the purpose of managing oral hygiene, including oral function, in older persons is to maintain and improve nutrition and QoL [54]. In addition, a study in India reported that chewing function is important despite the changes in oral function that occur with aging, and that the oral cavity should continue functioning even if the number of teeth that can occlude decreases [55]. A study in the USA reported that because the entire body and the oral cavity are closely related, maintenance of oral hygiene, including oral function, is reportedly necessary for maintaining systemic health [56].

This study has several limitations. First, the facilities surveyed in this study were affiliated with members of the Japanese Society of Geriatric Dentistry, and thus bias may exist in the sampling of facilities and participants. Second, this was a cross-sectional study, which impeded establishment of a causal relationship between the decrease in the number of occlusions and undernutrition in terms of BMI. Future longitudinal studies are warranted to elucidate the effects of restoring occlusal support with interventions such as dentures. Third, a history of neoplastic disease is reportedly related to BMI [57]. However, no significant association was observed between malnutrition and neoplastic disease among the participants with neoplastic disease in this study because only participants who were in remission for >5 years were included. In addition, the location of the tumor was not taken into account, and participants with a history of neoplastic disease at a site unrelated to nutrition may have influenced the results. It has been reported that depression is not associated with BMI [58], and the results of this study support this claim. However, a history of depression was a precursor to dementia in most cases in the present study, and we believe it was appropriate to consider it a part of the CDR. Fourth, depending on the condition of the elderly, physical function may interact with nutrition to improve or worsen the clinical condition. For example, this study did not take into account the possibility that sarcopenia in the elderly is associated with not only a decrease in whole-body muscle mass but also swallowing disorders and a decline in oral function. It has been reported that malnutrition is defined by factors other than BMI and reduced physical activity, such as activities of daily living (meal-related activities) and sarcopenia [59,60]. The present study did not consider such factors, and future studies should address this limitation.

In an aging society, the conditions of older persons are expected to become more diverse, and the need for assessment of nutrition and oral hygiene management for older persons is expected to increase in the future. We believe that the results of this study will contribute to the development of measures for maintaining and improving QoL of the elderly in nursing care settings in the future. Moreover, a decrease in the number of occlusal supports in elderly people who have lost their appetite because of aging or disease can lead to further malnutrition, creating a vicious cycle. The relationship between the overall condition and occlusal support was not clear in this study, and we believe the results can be strengthened by investigation of the causal relationship in future studies.

## 5. Conclusions

Our findings demonstrated that the decreased molar occlusions were associated with lower nutritional status in terms of BMI among Japanese older persons requiring long-term care in long-term care insurance facilities. Our findings suggest that maintaining occlusal support may help maintain nutritional status in older persons requiring long-term care. Additionally, our findings indicate that maintenance and restoration of occlusal support in regular dental management is an effective intervention to reduce weight loss in older persons requiring long-term care who are at high risk of undernutrition.

## Figures and Tables

**Table 1:** Characteristics of study participants and comparison between the BMI ≥ 20 kg/m^2^ and BMI < 20 kg/m^2^ groups.

	Total (n = 893)	BMI ≥ 20 kg/m^2^ (n = 453) (50.7%)	BMI < 20 kg/m^2^ (n = 440) (49.3%)	*p*-Value
	Mean ± SD, n (%) Median [Q1, Q3]	Mean ± SD, n (%) Median [Q1, Q3]	Mean ± SD, n (%) Median [Q1, Q3]	
Age (years)	86.8 ± 7.9	86.6 ± 8.2	87.0 ± 7.7	0.49
Sex (female), n (%)	719 (80.5)	369 (81.5)	350 (79.5)	0.499
Barthel Index	30 [7.5, 50]	30 [10, 52.5]	25 [5, 45]	<0.001
Clinical Dementia Rating				<0.001
0, 0.5	94 (10.5)	48 (10.6)	46 (10.5)	
1	124 (13.9)	80 (17.7)	44 (10.0)	
2	239 (26.8)	131 (28.9)	108 (24.5)	
3	436 (48.8)	194 (42.8)	242 (55.0)	
OHM				0.074
With requirements for OHM	529 (59.2)	263 (58.1)	266 (60.5)	
With requirements for OHM, but not implemented	173 (19.4)	80 (17.7)	93 (21.1)	
No requirements for OHM	191 (21.4)	110 (24.3)	81 (18.4)	
Number of molar occlusions				<0.001
0	290 (32.5)	114 (25.2)	176 (40.0)	
1	63 (7.1)	36 (7.9)	27 (6.1)	
2	63 (7.1)	39 (8.6)	24 (5.5)	
3	58 (6.5)	33 (7.3)	25 (5.7)	
4	419 (46.9)	231 (51.0)	188 (42.7)	
Nutritional intake method				<0.001
Regular diet	529 (59.2)	330 (72.8)	199 (45.2)	
Dysphagia diet	321 (35.9)	114 (25.2)	207 (47.0)	
Enteral nutrition	43 (4.8)	9 (2.0)	34 (7.7)	
Dysphagia				<0.001
Dysphagia	420 (47.0)	172 (38.0)	248 (56.4)	
Possibility of dysphagia	181 (20.3)	119 (26.3)	62 (14.1)	
No dysphagia	292 (32.7)	162 (35.8)	130 (29.5)	
Medical history				
Pneumonia	81 (9.1)	24 (5.3)	57 (13.0)	<0.001
Stroke	283 (31.7)	143 (31.6)	140 (31.8)	0.94
Diabetes mellitus	129 (14.4)	70 (15.5)	59 (13.4)	0.39
Respiratory disease	100 (11.2)	46 (10.2)	54 (12.3)	0.34
Cardiovascular disease	320 (35.8)	170 (37.5)	150 (34.1)	0.30
Neoplastic disease	103 (11.5)	50 (11.0)	53 (12.0)	0.68
Parkinson’s disease	47 (5.3)	17 (3.8)	30 (6.8)	0.05
Neurological disease	19 (2.1)	8 (1.8)	11 (2.5)	0.49
Depression	54 (6.0)	24 (5.3)	30 (6.8)	0.4
Dementia	622 (66.7)	312 (66.2)	310 (67.2)	0.78

Note: *p* < 0.05 was considered statistically significant. Q1, first quartile; Q3, third quartile. Abbreviations: BMI, body mass index; OHM, oral hygiene management; SD, standard deviation.

**Table 2:** Binomial logistic regression with BMI ≥ 20 kg/m^2^ or BMI < 20 kg/m^2^ as the dependent variable.

	OR	95% CI		*p*-Value
		Lower Confidence Limit	Upper Confidence Limit	
Age	1.00	0.99	1.02	0.67
Sex (female)	0.80	0.55	1.17	0.25
Barthel Index	1.00	0.99	1.01	0.82
Clinical Dementia Rating				
0, 0.5	Reference			
1	0.57	0.32	1.01	0.05
2	0.74	0.44	1.25	0.27
3	0.78	0.45	1.35	0.37
OHM				
With requirements for OHM	Reference			
With requirements for OHM, but not implemented	1.02	0.70	1.49	0.90
No requirements for OHM	0.85	0.59	1.22	0.37
Number of molar occlusions				
0	Reference			
1	0.55	0.30	1.00	0.05
2	0.52	0.28	0.96	0.04
3	0.53	0.29	0.98	0.04
4	0.70	0.50	0.98	0.04
Nutritional intake method				
regular diet	Reference			
dysphagia diet	2.53	1.79	3.56	<0.001
enteral nutrition	4.13	1.78	9.61	<0.001
Dysphagia				
Dysphagia	Reference			
Possibility of dysphagia	1.13	0.78	1.63	0.53
No dysphagia	0.58	0.38	0.89	0.01
Medical history				
Pneumonia	1.71	0.99	2.95	0.06
Stroke	0.88	0.64	1.21	0.42
Diabetes mellitus	0.99	0.66	1.49	0.98
Respiratory disease	1.03	0.66	1.63	0.89
Cardiovascular disease	0.88	0.65	1.19	0.39
Neoplastic disease	1.21	0.77	1.89	0.41
Parkinson’s disease	2.12	1.09	4.11	0.03
Neurological disease	1.81	0.66	4.96	0.25
Depression	1.15	0.63	2.10	0.65

Note: *p* < 0.05 was considered statistically significant. Abbreviations: OR, odds ratio; CI, confidence interval; OHM, oral hygiene management.

## Data Availability

The data presented in this study are available on request from the corresponding author. The data are not publicly available due to ethical-legal restrictions imposed by the Ethics Committee at the Japanese Society of Gerodontology.
